# Supplementary figures and images for: Amoeboid cells undergo durotaxis with soft end polarized NMIIA
Source: eLife. 2024 Dec 13;13:RP96821. doi: 10.7554/eLife.96821 (PMC11643633; doi:10.7554/eLife.96821)

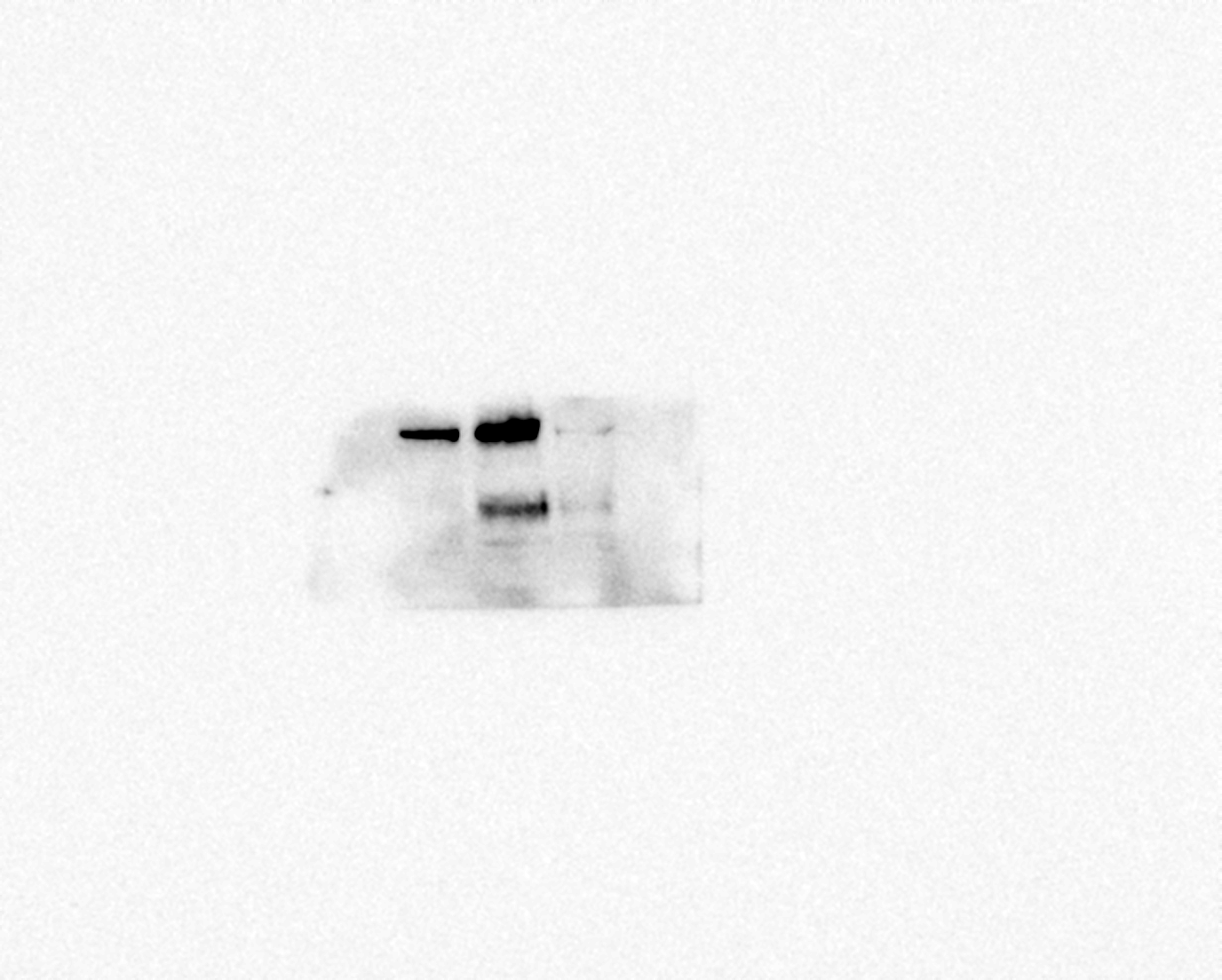

Supplement: Figure 3—source data 1. [file elife-96821-fig3-data1.zip › Figure 3-source data 1/NMIIA.tif]

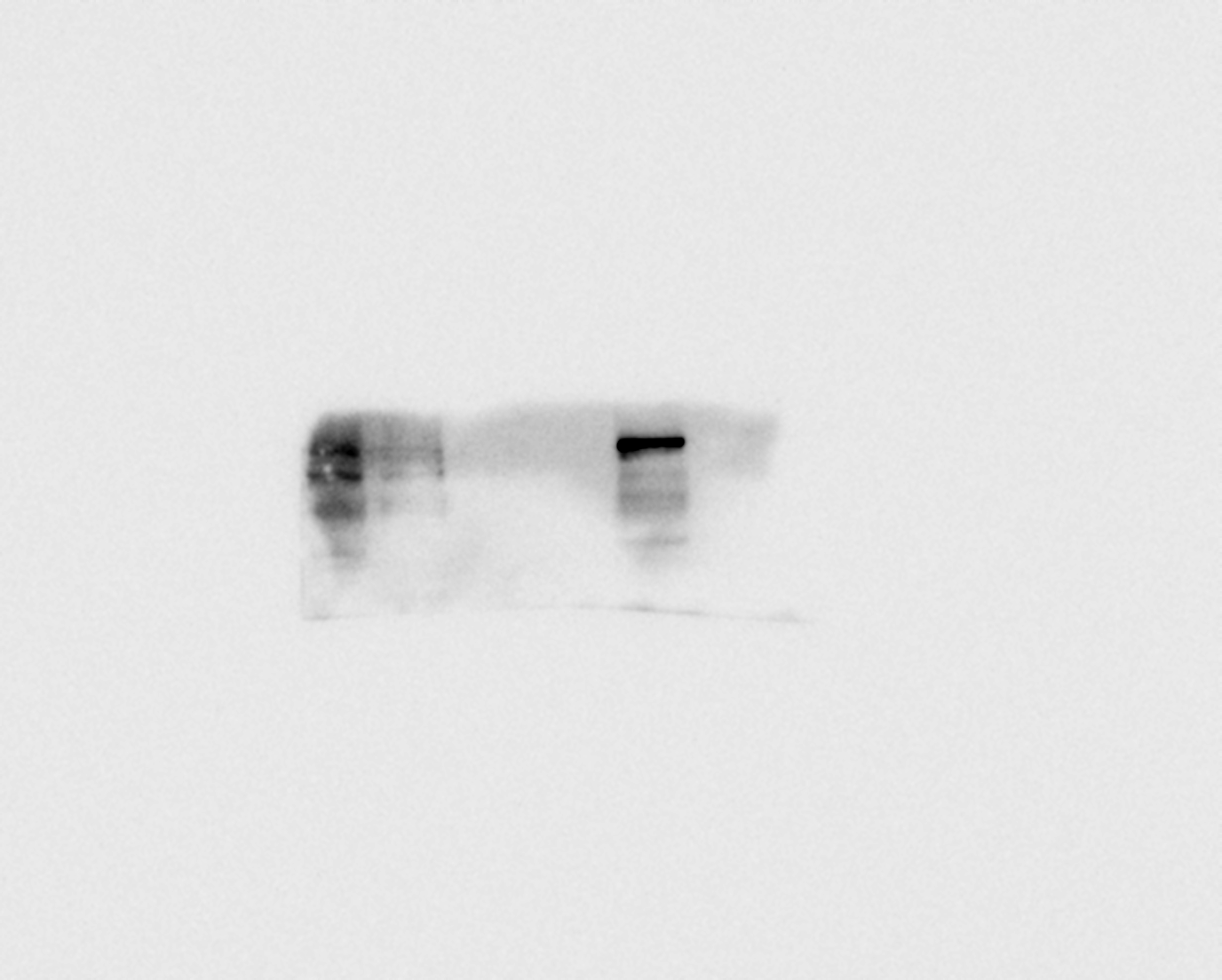

Supplement: Figure 3—source data 1. [file elife-96821-fig3-data1.zip › Figure 3-source data 1/NMIIB.tif]

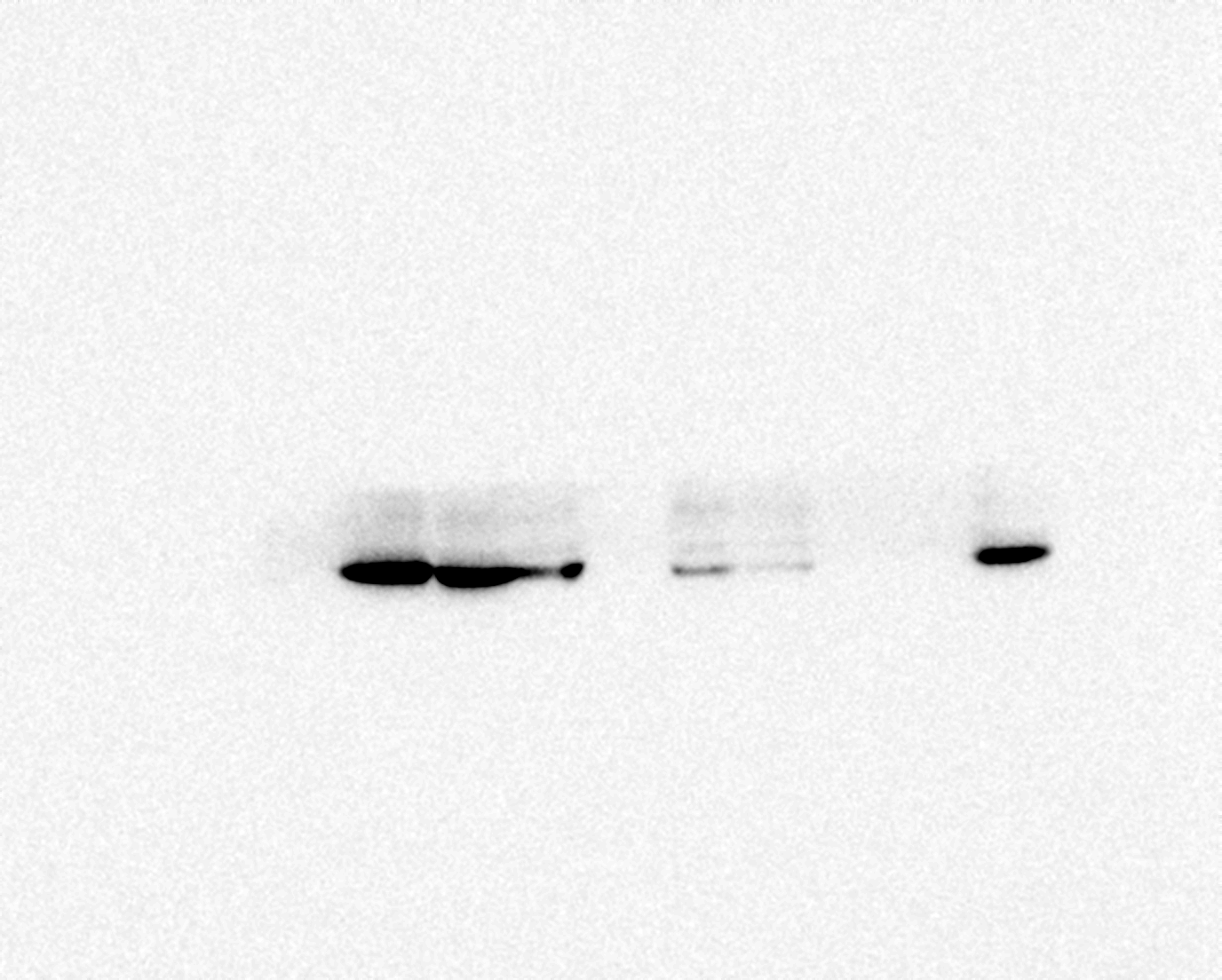

Supplement: Figure 3—source data 1. [file elife-96821-fig3-data1.zip › Figure 3-source data 1/tubulin for NMIIA.tif]

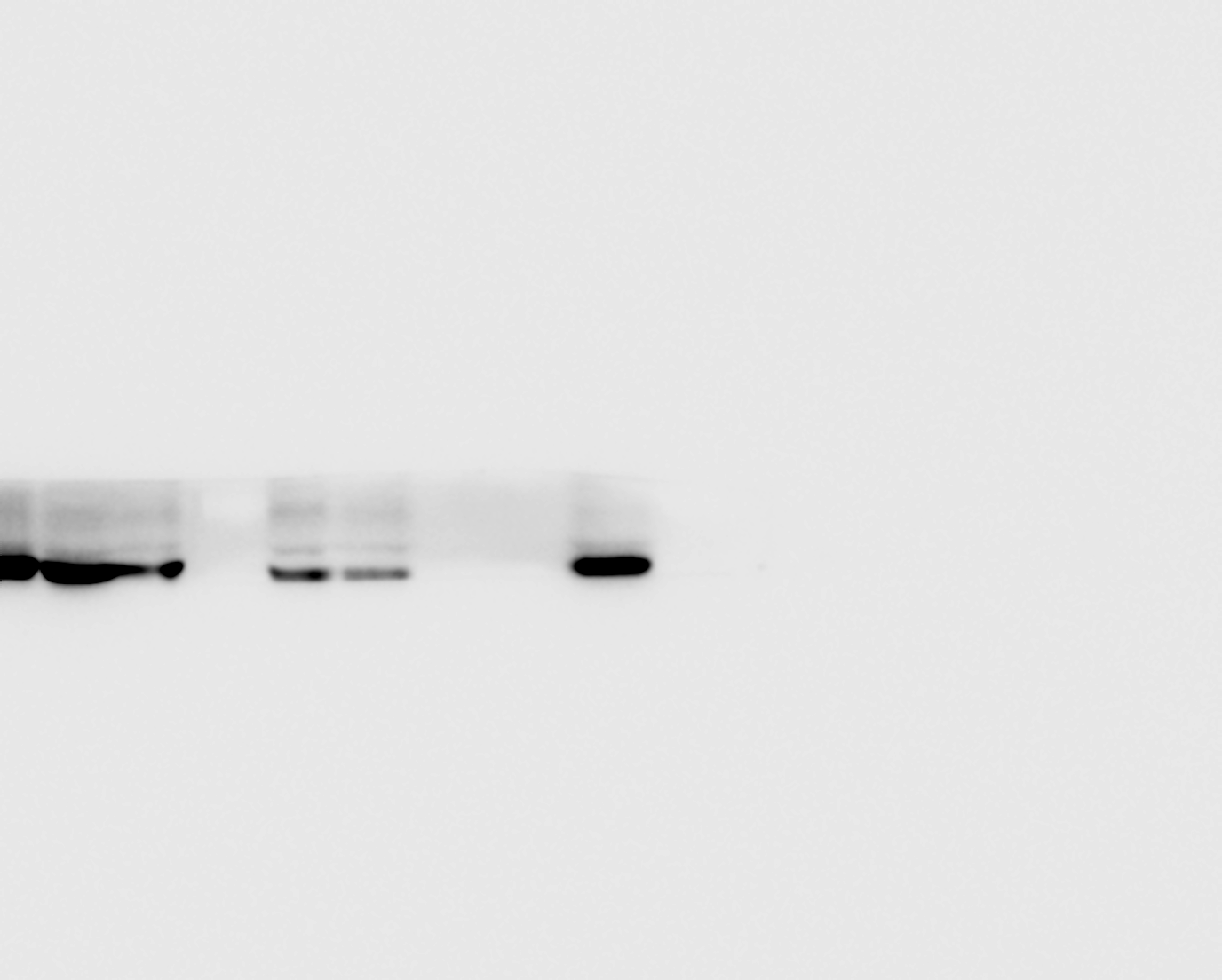

Supplement: Figure 3—source data 1. [file elife-96821-fig3-data1.zip › Figure 3-source data 1/tubulin for NMIIB.tif]

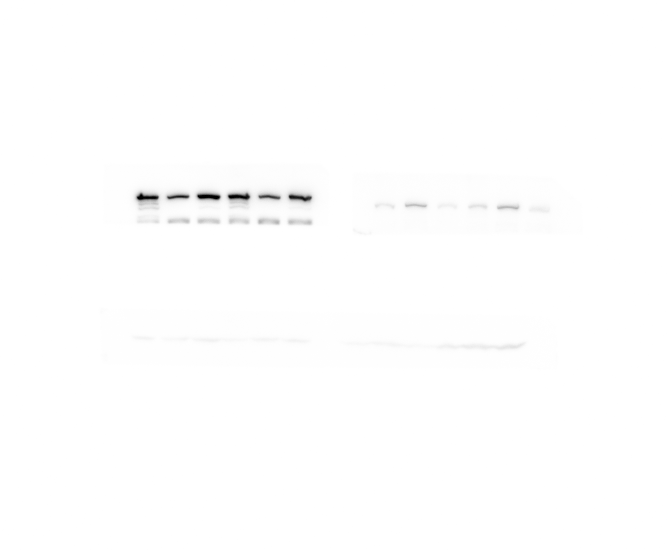

Supplement: Figure 3—figure supplement 1—source data 1. [file elife-96821-fig3-figsupp1-data1.zip › Figure 3-figure supplement 1-source data 1/NMIIA_1.tif]

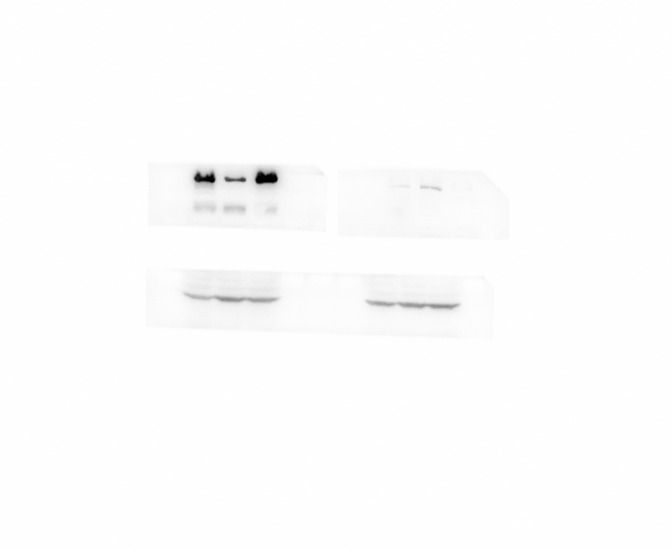

Supplement: Figure 3—figure supplement 1—source data 1. [file elife-96821-fig3-figsupp1-data1.zip › Figure 3-figure supplement 1-source data 1/NMIIA_2.tif]

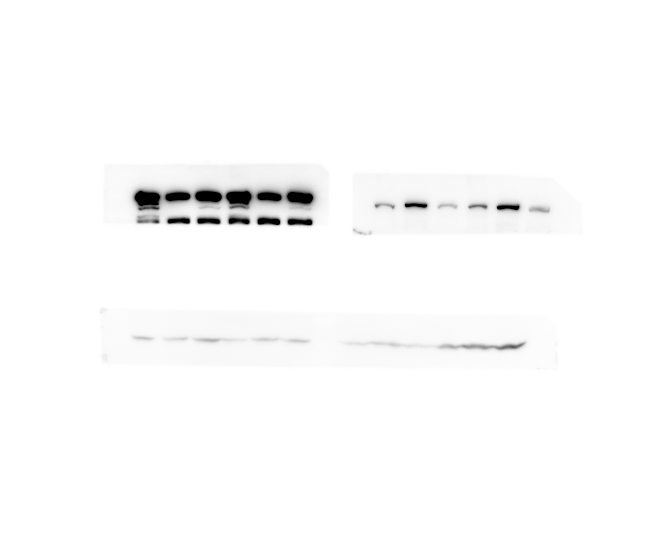

Supplement: Figure 3—figure supplement 1—source data 1. [file elife-96821-fig3-figsupp1-data1.zip › Figure 3-figure supplement 1-source data 1/NMIIB_1.tif]

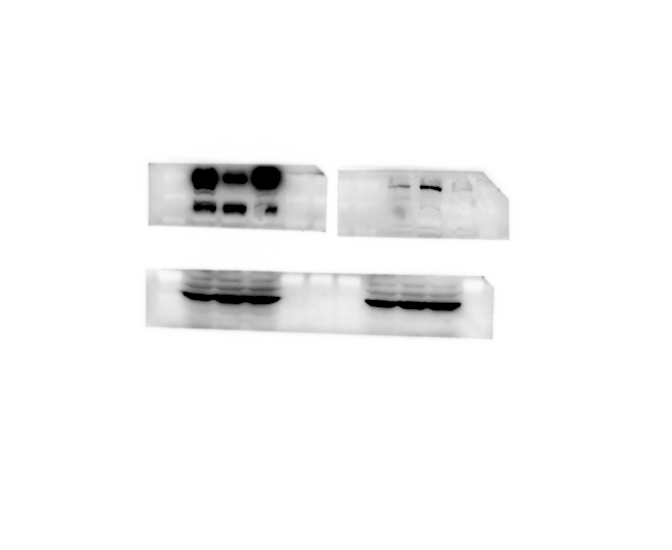

Supplement: Figure 3—figure supplement 1—source data 1. [file elife-96821-fig3-figsupp1-data1.zip › Figure 3-figure supplement 1-source data 1/NMIIB_2.tif]

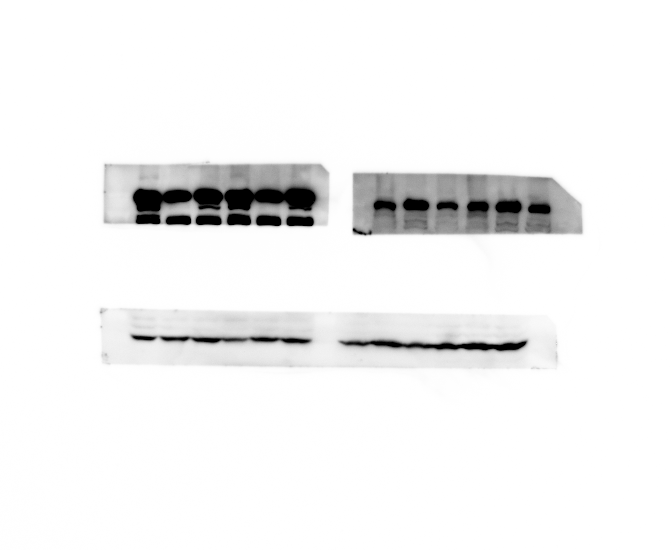

Supplement: Figure 3—figure supplement 1—source data 1. [file elife-96821-fig3-figsupp1-data1.zip › Figure 3-figure supplement 1-source data 1/tubulin_1.tif]

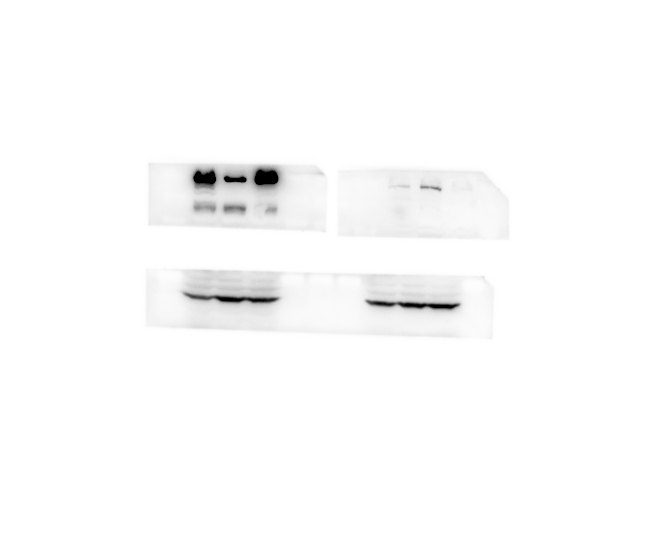

Supplement: Figure 3—figure supplement 1—source data 1. [file elife-96821-fig3-figsupp1-data1.zip › Figure 3-figure supplement 1-source data 1/tubulin_2.tif]

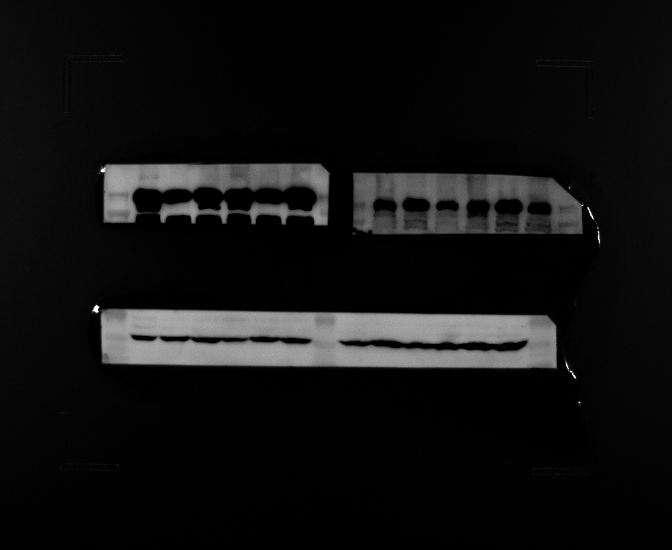

Supplement: Figure 3—figure supplement 1—source data 1. [file elife-96821-fig3-figsupp1-data1.zip › Figure 3-figure supplement 1-source data 1/Marker_1.tif]

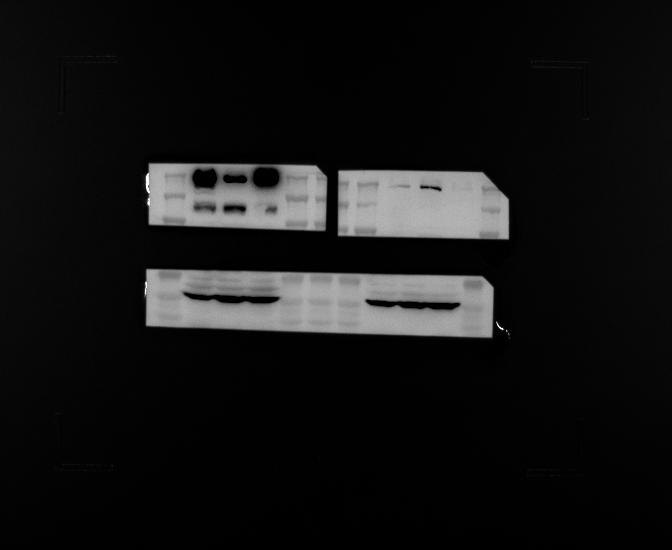

Supplement: Figure 3—figure supplement 1—source data 1. [file elife-96821-fig3-figsupp1-data1.zip › Figure 3-figure supplement 1-source data 1/Marker_2.tif]
