# Supplementary material for: Amoeboid cells undergo durotaxis with soft end polarized NMIIA
Source: eLife. 2024 Dec 13;13:RP96821. doi: 10.7554/eLife.96821 (PMC11643633; doi:10.7554/eLife.96821)

Figure 3-source data 2_western blot with labelled bands

NMIIA bands

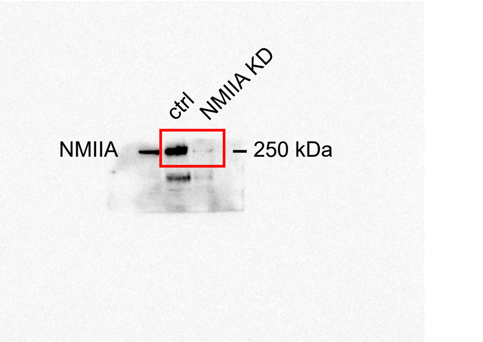

NMIIB bands

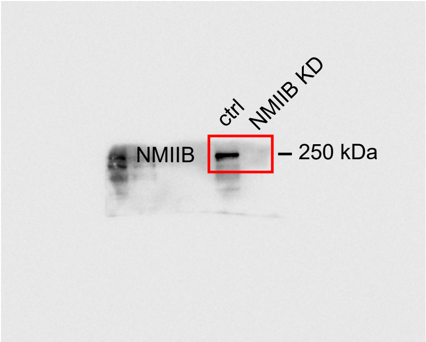

α-tubulin bands

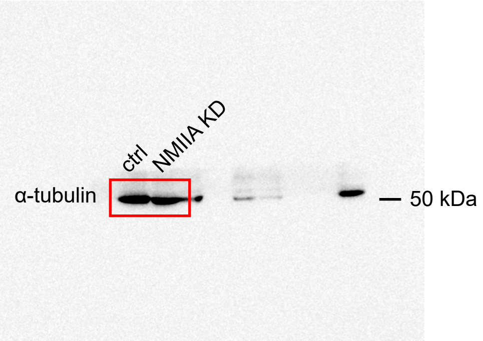

α-tubulin bands

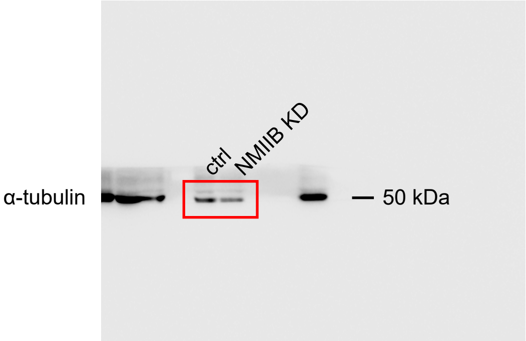

Supplement: Figure 3—source data 2. [file elife-96821-fig3-data2.zip › Figure 3-source data 2/Figure 3-source data 2_western blot with labelled bands.pdf]
