# Supplementary material for: Amoeboid cells undergo durotaxis with soft end polarized NMIIA
Source: eLife. 2024 Dec 13;13:RP96821. doi: 10.7554/eLife.96821 (PMC11643633; doi:10.7554/eLife.96821)
Supplement: Supplementary file 1. [file elife-96821-supp1.docx]

Table S1. Parameters used in simulations

| Model parameters | symbol | Physical value | Simulation value |
| --- | --- | --- | --- |
| Effective shear viscosity | $\eta$ | $1.67\text{ kPa}\cdot\text{s}$ | 5/3 |
| Effective elastic constant | $\kappa$ | $\text{2 nN}$ | 0.02 |
| Interfacial tension | $k$ | $\text{10 nN}\cdot\text{μm}^{6}$ | 0.1 |
| Shape factor | $\xi$ |  | 1.1 |
| Polymerization speed | $w$ | $0.03\text{ }\text{μm}/\text{s}$ | 0.0003 |
| Average density of actin | $c_{0}$ | $2\text{ μm}^{\text{-}3}$ | 2 |
| Phenomenological amplitude | $a$ | $10\text{ kPa}$ | 0.1 |
| Mobility parameter | $M$ | $0.1\text{ }\text{μm}^{2}/\left(\text{kPa}\cdot\text{s}\right)$ | 0.1 |
| Rotational viscosity | $\Gamma$ | $1\text{ kPa}\cdot\text{s}$ | 1 |
| Stability constant | $\alpha$ | $10\text{ kPa}$ | 0.1 |
| Fluid mass density | $\rho$ | $10^{14}\text{kg}/\text{m}^{3}$ | 10 |
| Rotational viscosity of myosin | $\Gamma_{m}$ | $5\text{ kPa}\cdot\text{s}$ | 5 |
| Reference substrate stiffness | $E_{0}$ | $1\text{ kPa}$ | 0.01 |
| Stiffness gradient | $E_{g}$ | $40\text{ }\text{kPa}/\text{mm}$ | 0.0004 |
| Basal diffusion rate of NMIIA | $D_{0}$ | $0.1\text{ }\text{μm}^{2}/\text{s}$ | 0.1 |
| Average concentration of NMIIA | $m_{0}$ | $1\text{ μm}^{\text{-}3}$ | 1 |
| Threshold of cell contractility | $\bar{\zeta}_{\text{thr}}$ | $0.1\text{ nN}\cdot\text{μm}$ | 0.001 |
